# How the COVID-19 Pandemic Is Reshaping the Trade Landscape and What to Do About It

**DOI:** 10.1007/s10272-020-0890-4

**Published:** 2020-06-07

**Authors:** Sébastien Jean

**Affiliations:** d’informations internationales (CEPII), Centre d’études prospectives et, 20, av. de Ségur, 75007 Paris, France

## Abstract

The COVID-19 pandemic carries heavy threats, and preserving stable and coordinated international trade relations will be essential to avoid catastrophic disorders or conflicts.

On 8 April 2020, the World Trade Organization (WTO) released its forecast for world trade in 2020, announcing that it expected a fall in volume of between 13% and 32%. Both the magnitude of the fall and the width of the forecasting range speak volumes about the violent blow the crisis dealt to international trade and about the uncertainty surrounding ensuing consequences. They reflect the deeply disruptive economic impacts of lockdown measures taken to counter the pandemic. Since these measures should be short-lived, part of their impact is temporary, and it is reasonable to expect that their removal will bring significant economic relief. Yet, I argue that this crisis will create lasting changes in the trade landscape and serious threats to the rules-based trading system, warranting a reconsideration of trade policy priorities in important respects.

## Four reasons why this crisis will bring about lasting change to the trade landscape

The fact that the shock is violent does not mean that its consequences will last. Four types of reasons lead to the expectation that some of them will be irreversible, though.

### Lasting changes in competitive positions and macroeconomic conditions

The looming economic crisis, expected to be of unprecedented scale in peace time at least for decades, will cause a large number of bankruptcies and buyouts, in a manner that is far from homogeneous across countries and sectors. While it is difficult to predict the size of the wave, it is likely to significantly alter competitive positions. Given the recognised importance of hysteresis and irreversibilities in international trade (e.g. Baldwin, 1990; Ramanarayanan, 2017), the consequences for international trade patterns might be long-lasting.

**Sébastien Jean**, Centre d'Études Prospectives et d'Informations Internationales; and National Research Institute for Agriculture, Food and Environment (INRAE), Paris, France.

The macroeconomic conditions will also likely suffer lasting alterations. Based on historical evidence from major pandemics, Jorda et al. (2020) conclude that significant macroeconomic effects persist for about 40 years, resulting in substantially depressed real rates of return, possibly as a result of increased risk aversion and precautionary saving. In the present case, this effect might reinforce the underlying trend of secular stagnation observed since the global financial crisis. The spectacular fall in inflation expectations witnessed in the US as early as March 2020 points in this direction, while China's factory prices fell at the sharpest rate in four years in April 2020 (e.g. Smith, 2020a; Smith 2020b; Chen and Lee, 2020). Another hint at such possible trends is the fact that upon the end of the strict lockdown, the Chinese economy witnessed a surge in exports in April but a decline in imports, suggesting that the negative consequences might be more long-lasting on demand than on supply. If confirmed, these trends suggest that the economic situation may be marked for some time by lagging demand. In this situation, firms' demand for protection from import competition is generally strong. While provisions exist within the WTO system to provide short-term relief in specific cases through safeguard measures, the risk that this pressure will result in spiralling tariffs and retaliatory measures is real.

### Exacerbated international tensions

The health crisis already spurred confused and non-cooperative responses, marked by more or less direct and formal export restrictions, including within the EU (Bown, 2020). According to the WTO (2020), in April 2020, 80 countries had introduced export prohibitions or restrictions as a result of the pandemic; these measures mostly concern health-related products, but some of them concern food products, even though there did not appear to be a supply shortage. Such measures can be very costly for foreign partners and heavily disruptive for international markets more generally, as previous episodes have shown, in particular in relation to food crises (e.g. Headey, 2011). Accordingly, they are bound to create strong tensions between partner countries.

This general context is aggravated by the mounting tensions between the US and China. While the 'phase one' deal signed in January 2020 marked a ceasefire in the trade war between the two countries, it did not resolve the underlying sources of tensions, far from it (Jean, 2020). Moreover, the pandemic will probably call into question China's enforcement of its import commitments under this agreement.

While the woeful absence of US leadership in the pandemic response can only add to the strategic rivalry between the two countries, increased tension is also visible in the trade arena, as witnessed by the US administration's tightening of the rules restricting exports of sensitive products to China.

## Increased risk aversion and reconsideration of global value chains

The coronavirus pandemic is a crisis of epic proportions, in health as well as in economic terms. As such, it will probably leave a lasting imprint upon perceptions. Since personal experience influences risk taking behaviours (e.g. Malmendier et al., 2011; Koudjis and Voth, 2014), it is likely that this crisis will increase risk aversion among a number of decision makers for a long time. This influence might lead to a reconsideration of efficiency-robustness trade-offs, potentially altering firms' international strategies. This has led to debates and questions surrounding vulnerabilities of global value chains (GVCs), and in various cases to calls to relocate production in order to boost resilience. As Miroudot (2020) emphasises, the capacity to maintain production during a crisis is referred to in the management literature as robustness, while resilience refers to the capacity to return quickly to normal operations after a crisis. And on both accounts, the vulnerability of GVCs is questionable compared to alternative, less international and less complex strategies, either in this crisis or in previous ones.

However, the most pressing questions raised by the coronavirus crisis about GVC vulnerabilities may not be related to resilience or even robustness, but rather to autonomy and control. Indeed, the pandemic shed crude light on the dependence of many advanced countries upon a very limited number of suppliers – frequently mainly upon China – to procure critical products such as Active Pharmaceutical Ingredients, reagents or even personal protective equipment. To assess the uncertainties created by such a situation, one has to take into account not only the risk of disruption of production in a given area (which, after all, is no less at home than abroad), but also threats like trade conflicts or disruption in the transportation sector. With rising international tensions, such dependence might increasingly be seen as a weakness. Firms might consider hedging this risk as well, which may lead them to re-assess their strategies, giving more weight to the benefit of diversified and easily controllable providers.

## Changes in the role of the state in the economy

From the state point of view, increased awareness of such situations of dependence raises concerns of strategic autonomy – understood in a broad sense – since it jeopardises its capacity to independently fulfil missions of vital importance. Since such issues were already high on the policy agenda before the coronavirus crisis, in particular in the EU, they are likely to trigger policy responses focused on domains deemed critical, to make sure the capacity to act forcefully is preserved even in conditions of crisis. For a long time, such concerns have been central to public policies in two areas: defence and food. They have translated into a different set of policies, warranting the special status of each of these domains in the international trading system and accommodating wide-ranging state intervention well beyond what is customary in other tradable sectors. But the COVID-19 pandemic has brought two additional domains to the front of strategic importance: health and digital infrastructure. Given the extraordinary tensions witnessed around procurement of, among other things, face masks and test reagents, there is probably no need to elaborate on the strategic status gained by the health sector. But it is worth emphasising that digital infrastructure also demonstrated its strategic importance, since it has been key to enabling the partial functioning capacities of many economies while maintaining fairly strict lockdown policies. This experiment will probably have lasting impacts on habits, norms and organisations, accelerating the increasing relevance of telepresence, a change with powerful disruptive potential (Baldwin, 2019). As heated controversies around 5G networks and the leading position of Huawei illustrates, the critical nature of the infrastructure upon which such new organisations and practices rely was already largely recognised. It can only be reinforced by the present crisis, warranting increased state attention on their autonomy in this area.

Beyond these concerns of strategic autonomy, states may also have to adjust their policies to citizens' heightened expectations with regard to the state's protective role against potential threats. To be sure, governments will be eager to make sure that they are better prepared to face any future health crises, but expectations and possible measures may extend well beyond this specific area. An important open question is to what extent it will transform perceptions and policies regarding climate change and, more generally, sustainable development. After all, even though a direct causation cannot be established in the present case, this pandemic can be viewed as a cautionary tale about the dangers for mankind of disrupting ecological equilibria.

Finally, the role of the state in the economy is already being altered in practice as a result of the support programmes carried out to prevent the pandemics and the ensuing lockdown measures from wreaking havoc on their productive systems. These programmes are extraordinary in their size and in the scope of the measures involved, including guaranteed loans, bailouts, direct or indirect subsidisation, with partial or complete nationalisations likely in several cases. They profoundly alter the practices in most economies and change the background against which disciplines have been considered and discussed for years, particularly in relation to industrial subsidies and state-owned enterprises (SOEs). As a matter of fact, several of these measures are probably in breach of commitments made in the WTO Agreement on Subsidies and Countervailing Measures (SCM). This is fully understandable given the circumstances, but it modifies significantly and, in all likelihood, for a long time, the background against which the highly sensitive question of industrial subsidies and SOEs will be discussed in the years to come.

## Objectives and principles for a European trade policy response

The bottom line is grim for the rules-based trading system: exacerbated tensions will make it all the more difficult to propose a coordinated response to the need to adapt public policies to the exceptional circumstances created by the COVID-19 crisis and to lasting pressures to protect domestic producers. Worse, these threats materialise in a context where the multilateral trading system was already destabilised not only by the ongoing trade war between the US and China but also, and more deeply, by its inability to address the structural challenges raised by the transformation of the world economy since the Marrakech Agreement was signed in 1994 (Jean, 2019). The pandemic and the ensuing structural changes can only add to the feeling that WTO rules have been conceived in a context that differs substantially from the one we are living in, increasing the risk of a loss of legitimacy. The rules-based trading system is threatened with irrelevance, and the inability of the WTO to play an active role in coordinating responses since the outbreak of the crisis does not help to assuage these concerns.

Lessons about the costs and dangers of disorderly responses to trade tensions had been learnt the hard way in the interwar period. Preserving coordination and stability in international trade relationships is thus more essential now than ever and should be the priority of any European trade policy response. It is, however, utterly challenging, both because of the frailty of the existing multilateral trading system and because of the necessity to make trade a lever, not an obstacle, to legitimise public objectives such as health, security and sustainable development. A rigid application of existing rules is unlikely to work, given their eroded legitimacy and the flaws already apparent in their enforcement. A multilateral agreement to reform the WTO would be the best solution that would allow for an updating of the rules, organising of mutually profitable grand bargains and coordinating of responses to global challenges. Unfortunately, it is clearly out of reach in the near future. A more pragmatic approach is needed, building upon political understandings and piecemeal reform, combining all available levels of political dialogue – at the WTO but also in the G20 and other international forums – so as to retain the trading system's consistency, but also to allow it more flexibility.

### Retaining consistency

The consistency of the multilateral trading system is currently jeopardised by the failure to maintain two of its overarching principles: transparency and enforceability. Safeguarding them is a priority.

Ensuring transparency requires that members live up to their notification obligations. Unfortunately, in many areas, notification obligations have been met with very long delays, when they have not been purely and simply ignored. This was the case, for instance, when the chair of the WTO Committee on Subsidies and Countervailing Measures told members at a meeting in 2016 that compliance with the obligation to notify subsidies "remains discouragingly low".[1] Judging by the recent WTO information on export-restrictive measures, the problem is far from being resolved: on 23 April, only 13 out of 46 members (counting the EU as one) had notified the WTO of the measures they introduced since the outbreak of the coronavirus crisis (WTO, 2020, 2). More political emphasis should be put on the necessity of accurate and timely notifications, as well as substantial deliberation, as preconditions for an orderly trading system.

The binding adjudication of trade dispute settlement, widely lauded as one of the main achievements of the WTO, is now paralysed by the US veto on the appointment of Appellate Body members. While the Multiparty Interim Arbitration Agreement is a poor substitute for this institution, it consists of several major players from the trading system, including the EU, China and Brazil. As such, it is a useful temporary solution to retain some enforceability, and hopefully to create positive dynamics, making it possible at some point to get the Appellate Body back to normal operation. Until then, it should be maintained and, to the extent possible, reinforced.

In addition to these cross-cutting principles, strengthening disciplines on export restrictions is another priority. While Article XI (§2.a) of the General Agreement on Tariffs and Trade (GATT) allows "export prohibitions or restrictions temporarily applied to prevent or relieve critical shortages of foodstuffs or other products essential", their use is especially costly for trading partners, particularly inflammatory in times of crisis, and rarely serves meaningful purposes. Agreement should be sought whereby exporters refrain from resorting to export restrictions,[2] even though the enforceability of such commitments is notoriously difficult to achieve because they tend to be used as emergency responses to crisis situations, in a temporality that is incompatible with rules-based dispute settlement, at least in the existing form.

1 See https://www.wto.org/english/news_e/news16_e/scm_28oct16_e.htm.

2 Evenett and Winters (2020) is an interesting proposal in this respect.

### Allowing flexibility

Given the intense pressure that the crisis has put on the multilateral trading system, improved flexibility is also required. The rules must not only be acceptable to stakeholders, they should also be consistent with vital public policy objectives such as health, security and sustainable development. This principle is already explicit in WTO agreements and, for instance, the objective of "allowing for the optimal use of the world's resources in accordance with the objective of sustainable development" is emphasised in the first paragraph of the preamble of the agreement establishing the WTO. Given the situation described above, however, it is fundamental to clarify the articulation between trade disciplines and other public policy objectives.

As mentioned above, the COVID-19 crisis has shed light on the specificity of critical products, and as a result, public policies are primarily guided by the need to respond to citizens' demands for protection and security. While defense and food products are already treated as exceptions for this reason, health-related products and digital infrastructures are not. Agreements leaving more space for state intervention for these products should be considered. Irrespective of opinions about the right policy response to these concerns, it should be recognised that organising public policies so as to secure due availability of the corresponding resources is up to each state and that the corresponding objectives have precedence on trade rules. This principle is actually consistent with GATT Article XX (General exceptions), stating inter alia that "nothing in this Agreement shall be construed to prevent the adoption or enforcement by any contracting party of measures: … (b) necessary to protect human, animal or plant life or health". However, the conditions of application of these general exceptions are very strict, making their use difficult in practice. The predictability and usability of these provisions could be greatly enhanced by coordination on the interpretation of what policies are authorised. An agreement specifically focusing on the limited scope of products concerned could even be sought to go beyond such interpretation, in particular in the case of health products.

The same logic should prevail with regards to policies tackling climate change. While responses to this existential challenge are second to none in importance, institutional asymmetries between subject areas have de facto created what Johnson (2015) calls the structural supremacy of trade law over environmental law (Bellora et al., 2020). This is increasingly troublesome, especially as global environmental challenges are looming larger. The rules on general exceptions and their interpretations should be clarified to make sure that efforts to combat climate change and promote sustainable development are not stymied by excessively rigid trade disciplines.

Finally, the changes entailed by the pandemic on the role of the state in the economy make it even more necessary than before to tackle the question of industrial subsidies and SOEs. This issue was already a central point of contention between big trading powers before the pandemic, with China as the main focal point, in a context marked by the lack of transparency and by the importance of subsidies in some industrial sectors (Jean and Nicolas, 2019). The sanitary crisis and ensuing massive public support plans are only adding to these concerns. In order to avoid a negative-sum competition game between subsidising programmes, a coordinated approach is urgent. An agreement, let alone new rules, is probably out of reach in the short term. However, enhancing transparency and deliberation might be a useful first step to at least assess the situation more accurately. Discussions about the respective merits and shortcomings of different types of public support could follow. The public support programmes being carried out in response to the coronavirus crisis are not going to vanish overnight as the present exceptional situation is here to stay at least for some time. Seeking agreed rules intended to minimise their distortive impacts is probably the most pragmatic approach to preserve some coordination in this area. In the longer term, it is the articulation between state and market that needs to be reconsidered in the multilateral trading system, but this is a daunting challenge that cannot be expected to be dealt with quickly.

## Preserving international trade relations

The COVID-19 pandemic carries heavy threats, and preserving stable and coordinated international trade relations will be essential to avoid catastrophic disorders or conflicts. The EU has an important role to play in defending the multilateral consistency, while also promoting flexibility. At least in an initial stage, damage control should be the main focus, but this crisis will also make it necessary to reconsider how trade disciplines should be articulated with other public policy objectives.
